# 5-Aminouracil and other inhibitors of DNA replication induce biphasic interphase–mitotic cells in apical root meristems of *Allium cepa*

**DOI:** 10.1007/s00299-020-02545-9

**Published:** 2020-04-23

**Authors:** Aneta Żabka, Konrad Krajewski, Justyna Teresa Polit, Joanna Bernasińska-Słomczewska, Janusz Maszewski

**Affiliations:** 1 Department of Cytophysiology, Faculty of Biology and Environmental Protection, University of Lodz, Pomorska 141/143, 90-236 Lodz, Poland; 2 Department of Molecular Biophysics, Faculty of Biology and Environmental Protection, University of Lodz, Pomorska 141/143, 90-236 Lodz, Poland

**Keywords:** *Allium cepa*, 5-AU, IM cells, Replication stress, ROS, Metabolic activity

## Abstract

**Key message:**

Induction of biphasic interphase–mitotic cells and PCC is connected with an increased level of metabolism in root meristem cells of *Allium cepa*.

**Abstract:**

Previous experiments using primary roots of *Allium cepa* exposed to low concentrations of hydroxyurea have shown that long-term DNA replication stress (DRS) disrupts essential links of the S–M checkpoint mechanism, leading meristem cells either to premature chromosome condensation (PCC) or to a specific form of chromatin condensation, establishing biphasic organization of cell nuclei with both interphase and mitotic domains (IM cells). The present study supplements and extends these observations by describing general conditions under which both abnormal types of M-phase cells may occur. The analysis of root apical meristem (RAM) cell proliferation after prolonged mild DRS indicates that a broad spectrum of inhibitors is capable of generating PCC and IM organization of cell nuclei. These included: 5-aminouracil (5-AU, a thymine antagonist), characterized by the highest efficiency in creating cells with the IM phenotype, aphidicolin (APH), an inhibitor of DNA polymerase α, 5-fluorodeoxyuridine (FUdR), an inhibitor of thymidylate synthetase, methotrexate (MTX), a folic acid analog that inhibits purine and pyrimidine synthesis, and cytosine arabinoside (Ara-C), which inhibits DNA replication by forming cleavage complexes with topoisomerase I. As evidenced using fluorescence-based click chemistry assays, continuous treatment of onion RAM cells with 5-AU is associated with an accelerated dynamics of the DNA replication machinery and significantly enhanced levels of transcription and translation. Furthermore, DRS conditions bring about an intensified production of hydrogen peroxide (H_2_O_2_), depletion of reduced glutathione (GSH), and some increase in DNA fragmentation, associated with only a slight increase in apoptosis-like programmed cell death events.

## Introduction

The dynamics of plant growth and development depends largely on the mitotic activity of root and shoot apical meristems (RAMs and SAMs), which provide cells for organogenesis and tissue expansion. Hence, any biotic, abiotic, or anthropogenic stress that affects these zones may exert a destructive effect on their functional organization and consequently may negatively influence the rate of vegetation. However, in order to reproduce, plants need to keep the ability to grow irrespective of the variety of detrimental events. Therefore, the surveillance activities used by plants must somehow combine the ‘stability’ that preserves species-specific modes of growth and differentiation with the ‘flexibility’ that can overcome harmful conditions. A detailed insight into molecular processes involved in the cell cycle control systems (also known as cell cycle checkpoints) and in the adaptation to stress (developed as the survival strategy) is generally thought to be indispensable for the understanding of the mechanisms underlying stress-resistance in plants (Cools et al. 2011).

Roots are endangered by harsh water conditions, such as increased salinity, desiccation, flooding, unfavorable pH values, or soil polluted with heavy metals, and are usually more severely affected by exposure to multiple abiotic stresses than the aerial parts of a plant (Franco et al. 2011). Additionally, all the growth-limiting factors are also capable of inducing reactive oxygen species, (ROS; Choudhury et al. 2017) which depending on the biological context, function as important signaling molecules and, at higher levels, as sources of numerous oxidative events leading to genomic lesions and to the ensuing disintegration of nuclear DNA. Therefore, an increase in ROS is not only the first defense line to help control environmental risks, but is also the main cause of damage at the chromosomal level. Furthermore, single- and double-stranded DNA breaks (SSBs and DSBs), which are among the most genotoxic insults, can arise spontaneously due to the defects in the S-phase of the cell cycle. Exposures to all kinds of chemical or physical agents that interfere with the replication machinery may thus lead to faulty nuclear divisions, causing cells either to lose their meristematic identity and to initiate vascular differentiation, or eventually (as a result of excessive levels of DNA damage), to employ an in-built mechanism for cellular destruction via apoptosis-like programmed cell death (AL-PCD) (Cools et al. 2011; Waterworth et al. 2011). It is essential, therefore, that we have a clear understanding of how root tip cells are preserved by triggering (but also by skipping) cell cycle checkpoints, which block DNA replication and mitotic activity as long as the stress endures.

The precise control over the sequence of events in interphase and mitosis largely depends on the functions of surveillance mechanisms that recognize various types of DNA damage and act in multiple ways to maintain genome integrity. The overall role of these molecular systems is to detect DNA replication blocks, SSBs, DSBs, or abnormally structured DNA, and to slow or arrest cell cycle progression; thereby, allowing time for appropriate repair mechanisms needed to correct genetic defects before they are passed on to the next generation of cells or organisms. Cells monitor S-phase progression and respond to DNA stress by using three partially distinct, but overlapping S-phase checkpoints: (1) the replication checkpoint, activated by stalled replication forks (RFs) to prevent DNA synthesis from new initiation sites (unfired origins), (2) the intra-S-phase checkpoint, turned on by the DNA damage during S-phase, and (3) perhaps the most remarkable control pathway conserved across all eukaryotic forms of life, the S–M checkpoint, which ensures that mitosis is not initiated in cells undergoing DNA replication (Eykelenboom et al. 2013). Additionally, by slowing down or inhibiting DNA synthesis, the S–M control system ensures that only a reduced pool of deoxynucleotide triphosphates (dNTPs) is required; thus, indirectly precluding the appearance and processivity of new RFs (Segurado and Tercero 2009; Cools et al. 2011). Failure of the S–M checkpoint leads to mitotic catastrophe of cells with incompletely replicated DNA (Stauffer et al. 2007).

There is a variety of well-known conditions or obstacles, which may result in slowing down or stalling of RFs progression, both referred to as the replication stress (RS). These comprise DNA nicks and gaps, stretches of single-stranded DNA (ssDNA), unrepaired DNA lesions, misincorporation of ribonucleotide triphosphates (rNTP), unusual DNA structures (e.g., hairpins, triplexes), dormant replication origins and many other *cis* and *trans* molecular barriers, including early-replicating and common fragile sites (ERFSs and CFSs), repetitive DNA elements, and collisions between the replication and transcription complexes (Magdalou et al. 2014; Mazouzi et al. 2014; Zeman and Cimprich 2014; Gelot et al. 2015; Berti and Vindigni 2016). Moreover, the list of RS-inducing factors has to be extended by the insufficient levels of proteins (such as histones and histone chaperones) and/or dNTPs. All these events can induce a set of cellular reactions, known as DNA damage response (DDR), which form a complex signaling network consisting of cell cycle checkpoints, DNA-repair mechanisms, and DNA damage tolerance pathways (Giglia-Mari et al. 2011).

The response of plant cells to RS is largely unexplored. Among many tools available to facilitate experimental studies on this subject, the most widely employed; thus far, was the use of hydroxyurea (HU), an inhibitor of ribonucleotide reductase (RNR). HU reduces the pool of dNTPs and affects replication fork progression (Saban and Bujak 2009). A number of side effects associated with HU-mediated inhibition of RNR comprise dissociation of the replication complex, accumulation of hemi-replicated intermediates, ssDNA interruptions at stalled RFs (which may then become converted into DSBs), formation of Holliday junctions through fork reversal, and other types of DNA damage, including those induced by ROS. Our previous studies focused on the effects of RS in onion (*Allium cepa*) primary root meristems exposed to low doses of HU clearly showed a definite interruption of the S–M checkpoint functions, leading cells either to premature chromosome condensation (PCC), or to an abnormal organization of chromatin, with both interphase (I) and mitotic (M) domains formed simultaneously on the opposing poles of the nuclei (IM cells; Żabka et al. 2010, 2012, 2013, 2015a, b). Consequently, our observations were the first to demonstrate a novel means by which interphase chromatin may become gradually compacted into mitotic chromosomes, creating large-scale compartments capable to perform independent functions relevant to their specific organizational designs. Furthermore, the recent screening studies suggest that the biphasic nuclear IM phenotype may also be induced in other crop species, e.g., sunflower (*Helianthus annuus*) and Legumes, such as broad bean (*Vicia faba*) and lentil (*Lens culinaris*; Żabka et al., in prep.).

In the present study, using *Allium cepa* root meristem model, we have shown that many inhibitors of DNA replication, when applied at low concentrations, may generate both PCC-like and biphasic IM cells similar to those formed under the prolonged (3-day) influence of HU. This type of response to the replication stress induced by 5-aminouracil (5-AU; selected for most of our further studies because of its highest efficiency in creating cells with the IM phenotype) has been found correlated with their significantly increased production of hydrogen peroxide (H_2_O_2_), depletion of reduced glutathione (GSH) and the enhanced levels of DNA synthesis, transcription and translation.

## Materials and methods

### Plant material

Calcium hypochlorite sterilized seeds of *Allium cepa* L. (obtained in 2018 from the agriculture farm “Lubiczow”) were sown on wet paper sheets in covered Petri dishes and germinated in the dark at 20 °C for 4 days. Seedlings with primary roots reaching 1.5 ± 0.2 cm were cultivated on blotting paper in trays filled with 10 mL of either distilled water (control samples) or chosen solutions of 5-aminouracil (5-AU; 750 μM) for 1, 3, 6, 12, 24, 48, and 72 h, and aphidicolin (APH; 750 μM), cytosine arabinoside (Ara-C; 100 μM), 5-fluorodeoxyuridine (FUdR; 0.74 μM), and methotrexate (MTX; 0.25 μM) for 72 h, at 20 °C in the dark.

### Feulgen staining

Excised roots were fixed in ice-cold Carnoy’s solution (absolute ethanol and glacial acetic acid; 3:1, v/v) for 1 h, washed several times with ethanol and, after rehydration, hydrolyzed in 4 M HCl (1 h). The staining procedure with Schiff’s reagent (pararosaniline) was performed according to the standard method (e.g., Polit et al. 2002). After rinsing in SO_2_–water (3 times) and distilled water, intensely stained apical segments (1.0–1.5 mm long) were cutoff, placed in 45% acetic acid and squashed onto Super-Frost microscope slides. Following freezing (dry ice), coverslips were removed, and the dehydrated slides were mounted in Canada balsam.

### EdU labeling and visualization of DNA replication on individual chromatin fibers

Onion seedlings were incubated with 10 µM 5-ethynyl-2′-deoxyuridine (EdU; Thermo Fisher Scientific) and 5-AU for 20 min, in the dark. Excised root tips were fixed in PBS-buffered 4% paraformaldehyde (4 °C; pH 7.4) for 40 min, and macerated for 15 min with citrate-buffered 2.5% pectinase from *Aspergillus niger* (Sigma), at pH 5.0. Meristems were squashed onto microscope slides (Polysine™, Menzel-Gläser) and air dried. After washing with PBS, DNA replication in nuclei were visualized using Click-iT DNA Alexa Fluor^®^ 555 Imaging Kit (Thermo Fisher Scientific), according to the supplier’s instructions. Slides were washed with PBS and stained with 15 μM 4′,6-diamidino-2-phenylindole (DAPI; Sigma-Aldrich) for 15 min, and, after washing with PBS, mounted in PBS/glycerol/DABCO mixture.

For visualization of DNA replication on individual chromatin fibers, onion root tips were incubated in EdU solution as described previously, fixed in Tris-buffered 1% formalin (4 °C; pH 7.2), and washed with ice-cold TRIS–HCl. Cell nuclei were isolated according to Van’t Hof (1980) by squeezing the meristems in a drop of PBS between two microscope slides, mixed with lysis buffer (0.5% sodium dodecyl sulfate, 50 mM EDTA, 200 mM Tris, pH 7.4), incubated at room temperature for 10 min and transferred onto the surface of coverslips silanized according to the modified protocol of Labit et al. (2008). The drop of extracted nuclear DNA was allowed to flow downwards by gravity and dried coverslips were processed for single staining using Click-iT^®^ EdU Alexa Fluor^®^ 555 reaction cocktail (Thermo Fisher Scientific) consisting of components prepared according to the vendor’s manual. Incubation was performed at 25 °C for 60 min. Slides were mounted in PBS/glycerol/DABCO mixture.

### 5-Ethynyl uridine incorporation and detection of nascent RNA

Control and 5-AU-treated onion seedlings were incubated for 1 h with 1 mM solution containing 5-ethynyl uridine (5-EU; Thermo Fisher Scientific), respectively with or without 5-AU, in the dark. Then, excised 1.5-mm-long root tips were fixed in PBS-buffered 4% paraformaldehyde (4 °C; pH 7.4) for 40 min, washed with PBS and macerated for 45 min with the citrate-buffered 2.5% pectinase (pH 5.0; 40 °C). After rinsing with cold PBS, root meristems were squashed onto microscope slides (Polysine™, Menzel-Gläser) in a drop of distilled water. Following freezing with dry ice, coverslips were removed, slides were washed with PBS, distilled water, and air dried. Macerated cells were then permeabilized with 0.5% Triton X-100 for 15 min. Nascent RNA was visualized using Click-iT^®^ RNA Alexa Fluor^®^ 488 Imaging Kit with the reaction cocktail (Thermo Fisher Scientific). After 1 h incubation at room temperature, slides were washed in Click-iT^®^ reaction rinse buffer and PBS, counterstained for 1 min with propidium iodide (PI; 0.3 mg mL^−1^) and washed in PBS. Specimens were mounted in PBS/glycerol/DABCO mixture.

### Protein synthesis assay

Nascent protein synthesis was detected using Click-iT^®^ HPG Alexa Fluor^®^ 488 Protein Synthesis Assay Kit (Thermo Fisher Scientific). Root fragments (1.5 cm long) cut off from the control, 5-AU-treated, and 50 μM cycloheximide-treated onion seedlings were transferred to 50 μM water solution of l-homopropargylglycine (HPG; control) or to the mixture of HPG + 5-AU or HPG + cycloheximide, in dark. After 30 min, apical root parts were fixed in phosphate-buffered saline (PBS)-buffered 3.7% paraformaldehyde (4 °C; pH 7.4) for 45 min. For maceration, excised meristems were rinsed twice in PBS and transferred for 45 min to the citrate-buffered 2.5% pectinase. Next, root tips were squashed onto microscope slides (Polysine™, Menzel-Gläser) in a drop of distilled water, and placed on dry ice. After 15 min, coverslips were removed, and the slides were washed with PBS, distilled water, and air dried. Permeabilization of macerated cells was performed using 0.5% Triton X-100 for 15 min. HPG incorporation was detected using Click-iT^®^ reaction cocktail consisting of components prepared according to the vendor’s manual. Incubation was performed at room temperature for 30 min and, after that slides were washed in Click-iT® reaction rinse buffer and PBS. Cell nuclei were stained for 5 min with DAPI at a concentration of 0.4 mg mL^−1^, washed in PBS and mounted in PBS/glycerol/DABCO mixture.

### Detection of H_2_O_2_

The generation of H_2_O_2_ was observed following treatment with 3,3-diaminobenzidine tetrachloride (DAB; Sigma-Aldrich) according to Thordal-Christensen et al. (1997), with minor modifications. Prior to fixation, control and 5-AU-treated onion seedlings were plunged in Tris-buffered (10 mM Tris, 10 mM Na_2_EDTA, 100 mM NaCl) DAB (1 mg/mL; pH 7.5) dissolved in distilled water (control) or 5-AU for 8 h. Excised root tips were fixed for 45 min (20 °C) in PBS-buffered 3.7% paraformaldehyde, washed several times with PBS, and placed in citrate-buffered 2.5% pectinase (pH 5.0, 37 °C for 45 min). Digested root meristems were washed with PBS and squashed onto microscope slides in a mixture of glycerol and PBS (9:1; v/v). Generated H_2_O_2_ was visualized under the microscope as a reddish-brown coloration of the cells (DAB reacts with H_2_O_2_ in the presence of peroxidase, forming a brown polymerized product).

### Detection of AL-PCD by TUNEL assay

For the terminal deoxynucleotidyl transferase dUTP nick end labeling staining (TUNEL assay), root meristems were fixed in PBS-buffered 4% paraformaldehyde for 20 min at 4 °C and stained using the in situ cell death detection kit (Click-iT^®^ TUNEL Alexa Fluor® 488 Imaging Assay; Thermo Fisher Scientific) according to manufacturer's instructions. Negative control was performed without the terminal deoxynucleotidyl transferase (TdT) and a positive control was performed with DNase I. Cell nuclei were stained with DAPI (as before) and mounted in a mixture of glycerol and PBS (9:1; v/v).

### Detection of glutathione using ThiolTracker™ Violet

Onion seedlings were incubated for 30 min with 20 µm ThiolTracker™ Violet (Thermo Fisher Scientific) dissolved in DPBS with C/M (Dulbecco’s phosphate buffered saline, containing Ca ++ and Mg ++ , glucose, and sodium pyruvate; Thermo Fisher Scientific). After washing with DPBS C/M, hand-made longitudinal sections of root meristems and RAM cells squeezed onto microscopic slides were counterstained with Hoechst 33342 dye (Sigma-Aldrich), mounted in a mixture of glycerol and PBS (9:1; v/v) and observed using a fluorescence microscope.

### DNA fragmentation assay by agarose gel electrophoresis

Total DNA was extracted and purified from excised onion root meristems grown in water (control) and treated with 5-AU for 72-h using the MagMAX™ Plant DNA Kit (Thermo Fisher Scientific). According to the procedure used earlier (Żabka et al. 2016), samples containing 10 μl of DNA and 2 μl of gel loading buffer (0.25% bromophenol blue, 30% glycerol in 1 × TAE composed of 40 mM Tris–HCl, 1 mM EDTA, 40 mM acetic acid, pH 8.0) were applied to 1% agarose gel in 1 × TAE buffer supplied with 1 mg/ml of ethidium bromide and separated by electrophoresis at 100 V for 3 h at room temperature. UV-fluorescent DNA fragments were photographed using a Gel UV Slider (Phoretix 1D image store system; Phoretix, England) in the Laboratory of Microscopic Imaging and Specialized Biological Techniques at the Faculty of Biology and Environmental Protection (University of Lodz).

### Microscopic measurements, observations, and analyses

Observations were made using E-600 epifluorescence microscope (Nikon) equipped with phase-contrast optics, U2 filter (UVB light; *λ* = 340–380 nm) for DAPI and Hoechst 33342, B2 filter (blue light; *λ* = 465–496 nm) for Alexa Fluor^®^ 488, or G2 filter (green light; *λ* = 540/25 nm) for Alexa Fluor® 555 and PI-stained cell nuclei. All images were recorded at exactly the same time of integration using a DS-Fi1 CCD camera (Nikon). Quantitative analyses and nuclear DNA fluorescence measurements were made after converting color images into greyscale and expressed in arbitrary units as mean pixel value (pv) spanning the range from 0 (dark) to 255 (white) according to described methods (Żabka et al. 2010, 2012). The obtained data were expressed as the mean values ± standard deviation of the mean (SD). Student's *t* tests for paired data were used to compare individual variables.

## Results

### Various inhibitors of DNA replication affect mitosis and induce biphasic IM nuclei in onion RAM cells

Considering the recognized effects of HU and its capacity to promote IM cells in onion root meristems (Żabka et al. 2010, 2012), our experiments were primarily aimed at establishing the extent to which similar outcomes may be produced by other drugs known to affect different aspects of DNA synthesis. As shown in Table 1, a spectrum of inhibitors efficient in generating biphasic (IM) organization of cell nuclei (tested over a broad range of dilutions) included: 5-aminouracil (5-AU), a thymine antagonist that blocks DNA synthesis and binds as a third strand capable of forming triplex DNA structures through Hoogsteen hydrogen bonds (Shaker et al. 2016), aphidicolin (APH), an inhibitor of DNA polymerase α (Yokoyama et al. 2016), 5-fluorodeoxyuridine (FUdR), an inhibitor of thymidylate synthetase (Anderson et al. 2016), methotrexate (MTX), a folic acid analog that inhibits purine and pyrimidine synthesis (Cutolo et al. 2001), and cytosine arabinoside (Ara-C), which incorporates into DNA and inhibits DNA replication by forming cleavage complexes with topoisomerase I (Wang et al. 2010). No induction of IM cells was detected in experimental series using excess of thymidine or 5-FU.

**Table 1:**
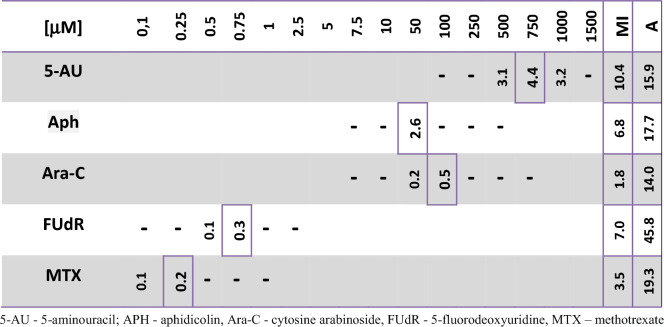
Maximum mean number of biphasic (IM) cell nuclei per root meristem (enframed), mitotic index (MI), and percent of aberrant mitotic divisions (A) in onion RAMs after 72-h treatment with selected inhibitors, tested over a range of dilutions (μM)

*5-AU* 5-aminouracil, *APH* aphidicolin, *Ara-C* cytosine arabinoside, *FUdR* 5-fluorodeoxyuridine, *MTX* methotrexate.

**Fig. 1:**
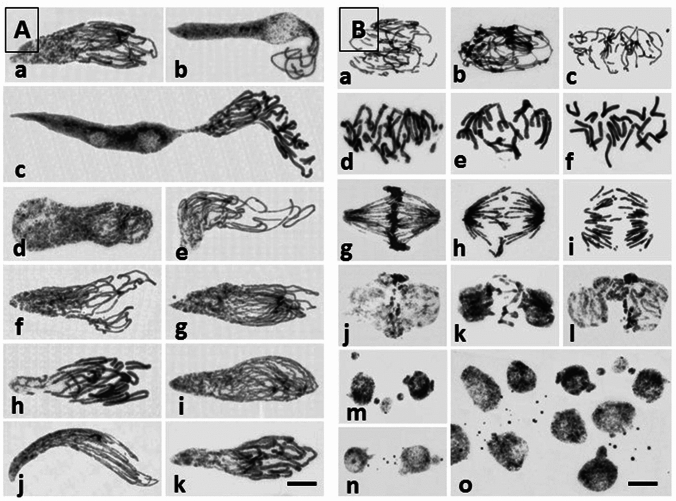
Nuclear Feulgen staining of aberrant mitotic cells in *A. cepa* RAMs. Scale bars = 20 μm. **A** A variety of biphasic IM cells formed after 72 h treatment with 5-AU (**a**–**c**); note the IM cell nucleus combining biphasic and amitotic phenotype (**c**), APH (**d**, **e**), Ara-C (**f**, **g**), FUdR (**h**, **i**), and MTX (**j**, **k**). **B** Selected cell nuclei with evident PCC-like morphology during prophase (**a**–**c**), metaphase (**d**–**f**), anaphase (**g**–**i**) and telophase (**j**–**l**) following treatment with 5-AU (**a**, **d**, **g**, **j**), Ara-C (**b**, **e**, **h**, **k**), and MTX (**c**, **f**, **i**, **l**). Post-telophase cell nuclei with micronuclei formed after treatment with APH (**m**) and FUdR (**n**). Numerous micronuclei in 5-AU-treated RAM cells (**o**)

Following 3-day mild treatments with 5-AU, APH, FUdR, MTX, and Ara-C, the morphological signatures of onion root apex meristem (RAM) cells were strongly consistent with those observed after the prolonged incubations using low concentrations of HU (Żabka et al 2010, 2012). All inhibitors produced a remarkable diversity of cells, with a dominant interphase fraction (visually normal) and a smaller population of mitotic cells displaying either unusual biphasic nuclear structures (IM cells; Fig. 1A) or evident symptoms of premature chromosome condensation (PCC-cells; Fig. 1B). In accordance with our earlier data (Żabka et al. 2012; 2015a), cell size seems to constitute one the major parameters determining either one or the other of the two abnormal types of nuclear division. The IM cell nuclei (also termed as “octopus-like” for their chromosomal arms sticking out of the interphase nuclear bodies) are restricted merely to a group to extremely long cells with major to minor axis ratios exceeding a factor of 3.0, whereas the PCC-like phenotype is confined to a group of M-phase cells approaching the maximum length to width ratio less than 2.5. A variety of gradients of chromatin condensation observed in the IM cell nuclei extended from interphase to early stages of prophase (Fig. 1A d, j), from interphase to late stages of prophase (Fig. 1A a, b, f, g, i), or from early to late stages of prophase (Fig. 1A e, h, k). In 5-AU-treated root meristems, some cells became extremely long with correspondingly elongated and bizarrely amitosis-like arranged IM nuclei (Fig. 1A c).

**Fig. 2:**
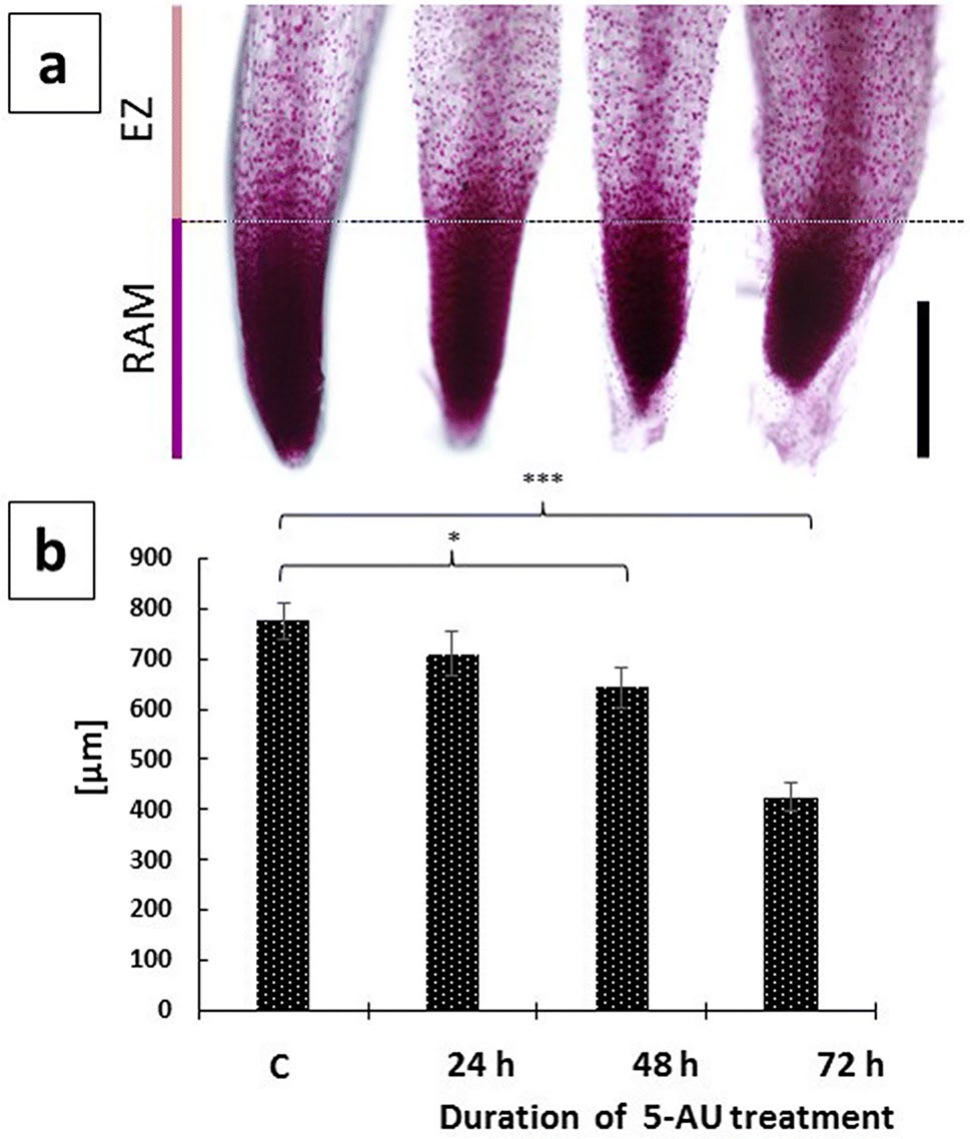
Root meristem shortening during continuous incubation of *A. cepa* seedlings with 5-AU. Feulgen-stained root tips aligned according to the transition regions between RAMs and elongation zones (EZ), demarcated by dotted line (**a**); corresponding RAM lengths [μm] in control plants (**c**) and after 24, 48, and 72 h treatment periods (**b**). Scale bar = 500 μm. When compared with the control, statistically significant changes in mean RAM length (± SD) are marked by asterisks: * indicates *p* < 0.05 and *** indicates 0.001

Despite no evident impact on the DNA cell cycle profiles (not shown) and a varied effect on the mitotic indices (ranging from an increase after incubation with 5-AU to a considerable decrease in plants exposed to Ara-C; Table 1), all treatments resulted in a significant rise in various types of chromosome disorders (Fig. 1B) and a substantial reduction in onion RAM size (illustrated by Fig. 2a, b, following incubations with 5-AU). In addition to entangled chromosomes in prophase (Fig. 1B a–c), only a relatively small fraction of metaphase cells (about 9%) revealed chromosomal gaps, breaks, and an abnormal arrangement of chromosomes in the equatorial plane of the cell (Fig. 1B d–f). Most frequently, structural aberrations (such as acentric fragments of chromosomes and chromosomal bridges) were observed in ana- (Fig. 1B g–i) and telophase cells (Fig. 1B j–l). In consequence, the 3-day incubation with each of the inhibitors enhanced the frequency of micronuclei (Fig. 1B m–o), with their most significant accumulation in 5-AU-treated roots (up to 240.7 ± 16.2, compared with 17.1 ± 4.3 in the control plants, both values per 1000 cells).

### Incorporation of EdU into nuclear DNA during 5-AU-induced replication stress

**Fig. 3:**
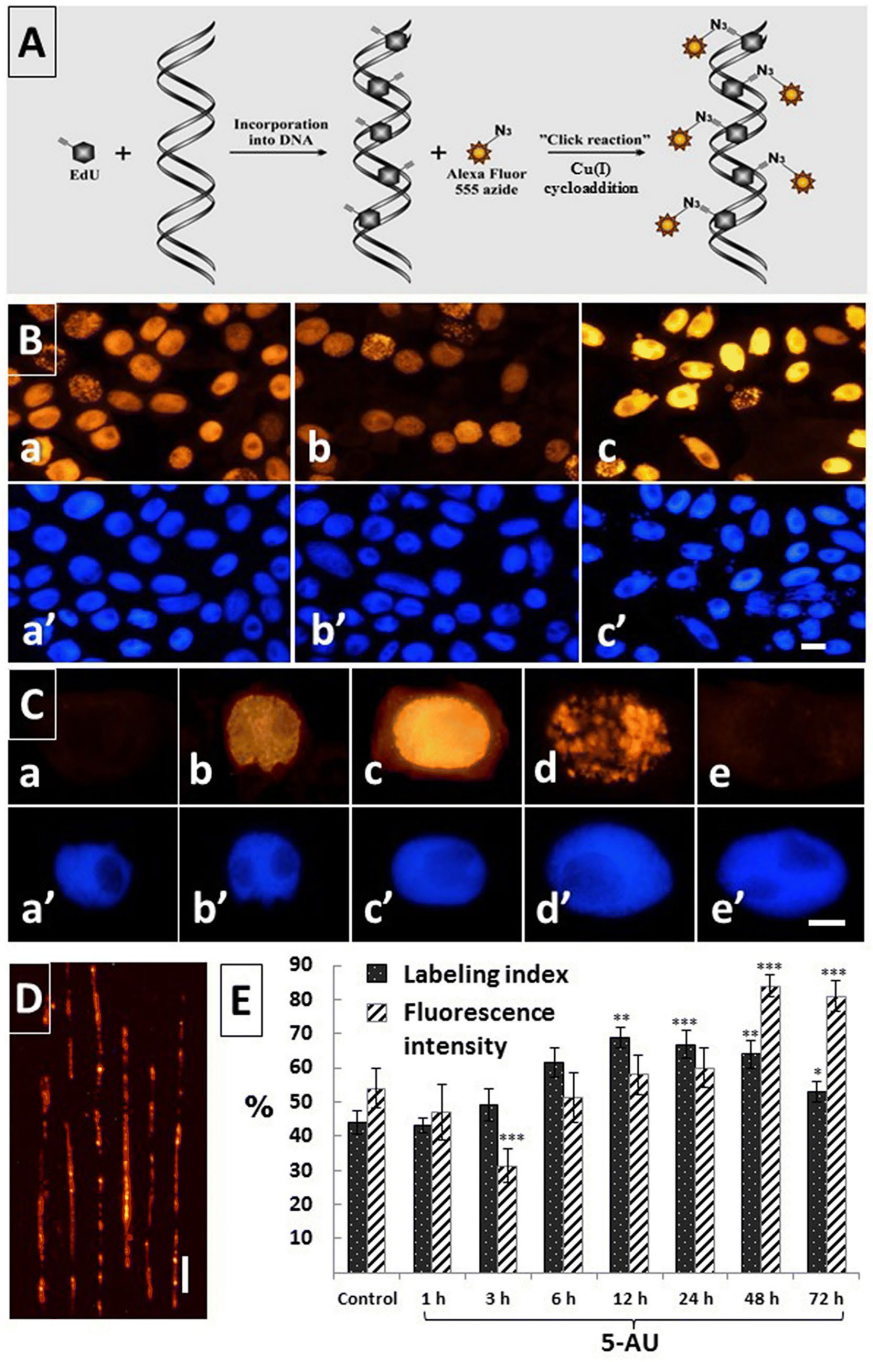
Analysis of DNA replication in *A. cepa* RAMs by EdU incorporation. **A** Schematic representation of copper(l)-catalyzed Alexa Fluor^®^ 555 azide-alkyne "click" reaction used to detect replicating DNA. **B** RAM cell populations labeled with EdU (**a**–**c**) and the same cells counterstained with DAPI (**a**–**c**) in the control seedlings (**a**, **a**), after 1 h (**b**, **b**) and 48 h (**c**, **c**) incubations with 5-AU. Scale bar = 20 μm. (C) EdU labeling of RAM cells at successive stages of interphase: G1 (**a**), early S (**b**), middle S (**c**), late S (**d**), G2 (**e**), with corresponding images of the same cells counterstained with DAPI (**a**–**e**). Scale bar = 10 μm. **D** Single molecule DNA fibers isolated from actively replicating cell nuclei after their 48 h treatment with 5-AU. Scale bar = 10 μm ≈ 20 kbp. **E** Mean percentage (± SD) of EdU-labeled cells (labeling index; dotted diagram) and mean (± SD) intensity of EdU labeling (Fluorescence intensity, lined diagram), calculated as a percentage of the maximum possible brightness (pixel value = 255) evaluated for selected groups of mid-S-phase cell nuclei (determined microfluorimetrically following DAPI staining). The data represent values obtained for the control and 5-AU-treated onion RAM cell nuclei at successive periods of incubation. When compared with the control, statistically significant changes in mean values (± SD) are marked by asterisks: * indicates *p* < 0.05, ** indicates *p* < 0.01, and *** indicates *p* < 0.001

The thymidine nucleoside analog 5-ethynyl-2′-deoxyuridine (EdU) was applied for the recognition of S-phase cell nuclei in both control and 5-AU-treated onion RAMs after successive 1-, 3-, 6-, 12-, 24-, 48-, and 72-h incubation periods (Fig. 3). Using this method, detection of DNA synthesis relies on the Cu^+2^-mediated covalent reaction between an alkyne of EdU and an Alexa Fluor^®^ 488 azide (Fig. 3A). The proportions of EdU-labeled (actively replicating) to EdU-unlabeled (non-replicating) cell nuclei are exemplified by fluorescence microscopy images taken from the control (Fig. 3Ba, a’) and 5-AU-treated RAM cell populations (after 1 and 72 h incubation periods, shown in Fig. 3Bb, b’ and Fig. 3Bc, c’, respectively) , and quantified as labeling indices in Fig. 3E. When compared with the control root cells, after the first 1-h treatment with 5-AU, a slight decrease in EdU-labeled cells was noted. Then (starting from the 3 h time point), the number of replicating cells increased, reaching the highest value (69%) at the 12 h time point. Beginning from the 24-h treatment, population of EdU-labeled cells gradually decreased to about 52% (72-h incubation period; Fig. 3E).

In order to evaluate the time-dependent changes in the dynamics of DNA replication, a similar set of data was collected in the sequential periods of 5-AU treatment by measuring EdU fluorescent labeling intensities, calculated as a percent of the maximum possible brightness (pixel value), represented on a grayscale range from 0 (black = 0%) to 255 (white = 100%; Fig. 3E). Considering natural variation in the amount of emitted fluorescence and in their spatial distribution patterns across chromatin at different stages of the S-phase (Fig. 3C), onion RAM cells were selected with respect to DNA C values corresponding to the middle S-phase ranging from 2.8 to 3.2C (where C is the amount of DNA in the unreplicated haploid genome). In consequence, at each time point of the study, the nuclei chosen for analysis were characterized by the strongest and most homogenous EdU labeling density (Fig. 3Cc).

When compared with the intensities of fluorescence estimated for the control mid-S-phase cell nuclei (Fig. 3Ba), the relevant data scored at the earliest measurement time points throughout continuous incubation with 5-AU (after 1 h, and significantly, after 3 h) were markedly lower (Fig. 3Bb, E). At subsequent periods (6 to 24 h), the dynamics of DNA replication increased considerably, reaching still higher values of fluorescence intensity to the maximum levels at 48 (Fig. 3Bc) and 72 h of treatment. An extremely strong EdU fluorescence signal emitted by mid-S-phase chromatin from 5-AU-treated onion RAM cell nuclei has enabled us to visualize an ongoing process of replication at a single DNA molecule level. As shown in Fig. 3D, owing to a high-fluorescence quantum yield, a simplified version of molecular combing technique using silane-coated glass slides provides both determination of origin density along the DNA and good separation of its fibers. To our best knowledge, positive results in experiments using EdU-labeled plant S-phase nuclei to demonstrate micropatterning along stretched and aligned DNA molecules has never been reported before in literature.

### 5-AU-induced changes in the dynamics of transcription and translation

**Fig. 4:**
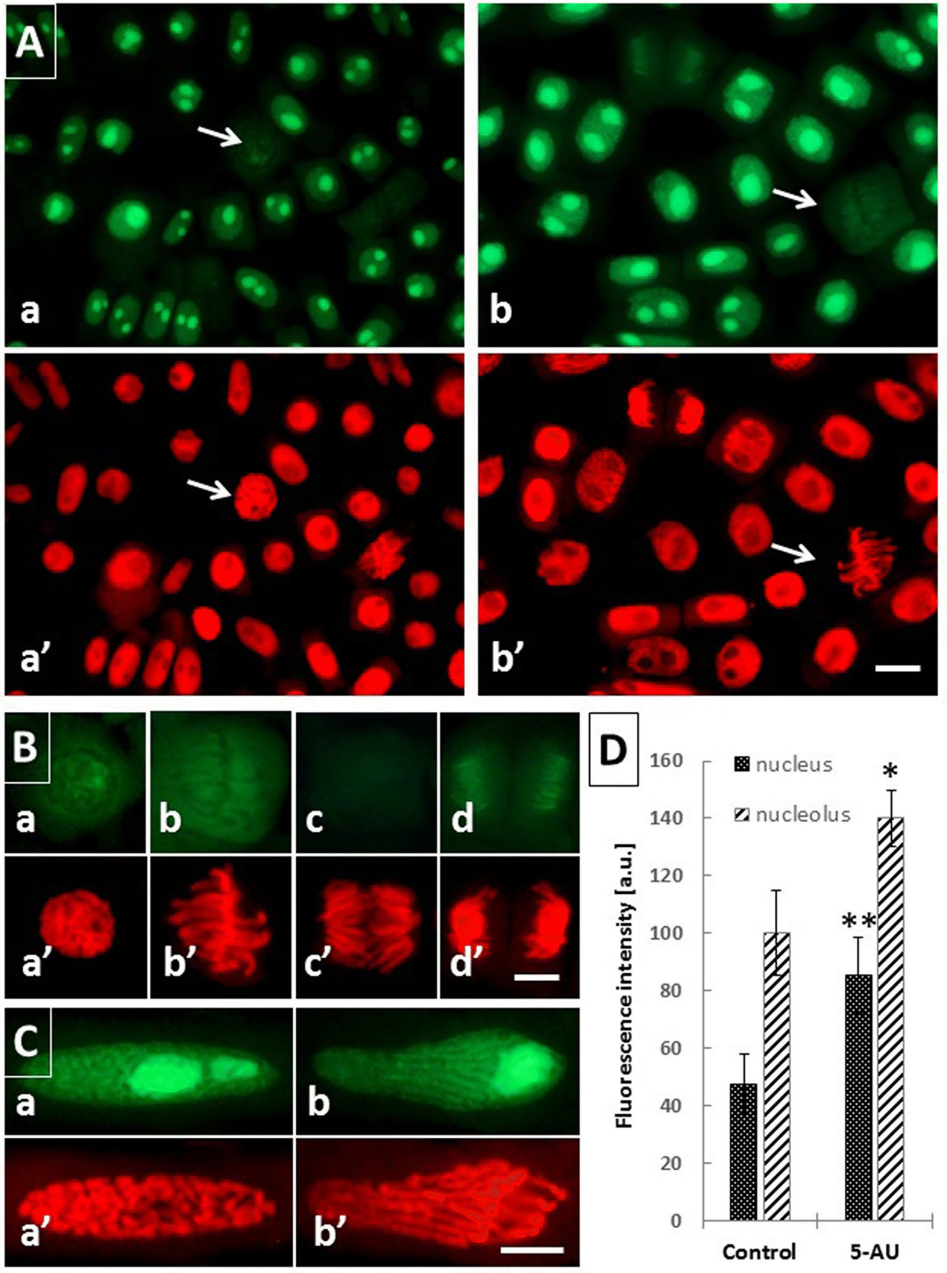
Analysis of nascent transcription level in *A. cepa* RAM cells using EU incorporation followed by Click-iT^®^ RNA Alexa Fluor^®^ 488 imaging. **A** Detection of RNA synthesis in the control root tip cells (**a**) and after 72 h 5-AU treatment (**b**); corresponding images of the same cells counterstained with PI (**a** and **b**); note faint fluorescence signal emitted in prophase (**a**, **a**; arrows) and metaphase cells (**b**, **b**; arrows). Scale bar = 20 μm. **B** Changes in nascent transcription level during successive stages of mitosis in the control onion cells at prophase (**a**), metaphase (**b**), anaphase (**c**), and telophase (**d**), with corresponding images of the same cells counterstained with PI (**a**–**d**, respectively). Scale bar = 10 μm. **C** Increased EU incorporation into nascent transcripts observed in elongated prophase (**a**) and the biphasic IM cells after 72 h treatment with 5-AU (**b**); corresponding images of the same cells counterstained with PI (**a**, **b**, respectively). Scale bar = 20 μm. **D** Microfluorimetric evaluation of mean nascent transcription levels measured for the nucleus and nucleolus in the control and 5-AU-treated onion RAM cells. When compared with the control, statistically significant changes in mean fluorescence levels (± SD) expressed in arbitrary units [a.u.] are marked by asterisks: * indicates *p* < 0.05 and ** indicates 0.005

Total transcriptional and translational activities were evaluated both in the control and 5-AU-treated RAM cells of *A. cepa* by using “click” chemistry technique and fluorometric measurements. The first assay, performed by incorporating the alkyne-modified uridine analog 5-ethynyl uridine (EU; Fig. 4), allows for a rapid detection of newly formed transcripts generated by RNA polymerases without the need of using radioactive precursors or antibodies. In general, the highest EU signals were observed in the nucleoli, which is attributed to an intense ribosomal RNA synthesis (Fig. 4Aa, b, Ca, b). When compared with the control RAM cells (Fig. 4Aa), incubation with 5-AU caused a considerable enlargement of the nucleoli (Fig. 4Ab) and about 40 per cent increase in the fluorescence intensity, noticed both in the nucleoplasmic and in the nucleolar regions (Fig. 4Ab, D). No significant differences in weak EU staining were observed between successive stages of mitosis in the untreated seedlings (Fig. 4B), whereas in the elongated prophase cells formed after the prolonged (72 h) 5-AU treatment, a significant fluorescence of RNA could be detected around and between the condensing chromosomes (Fig. 4C).

**Fig. 5:**
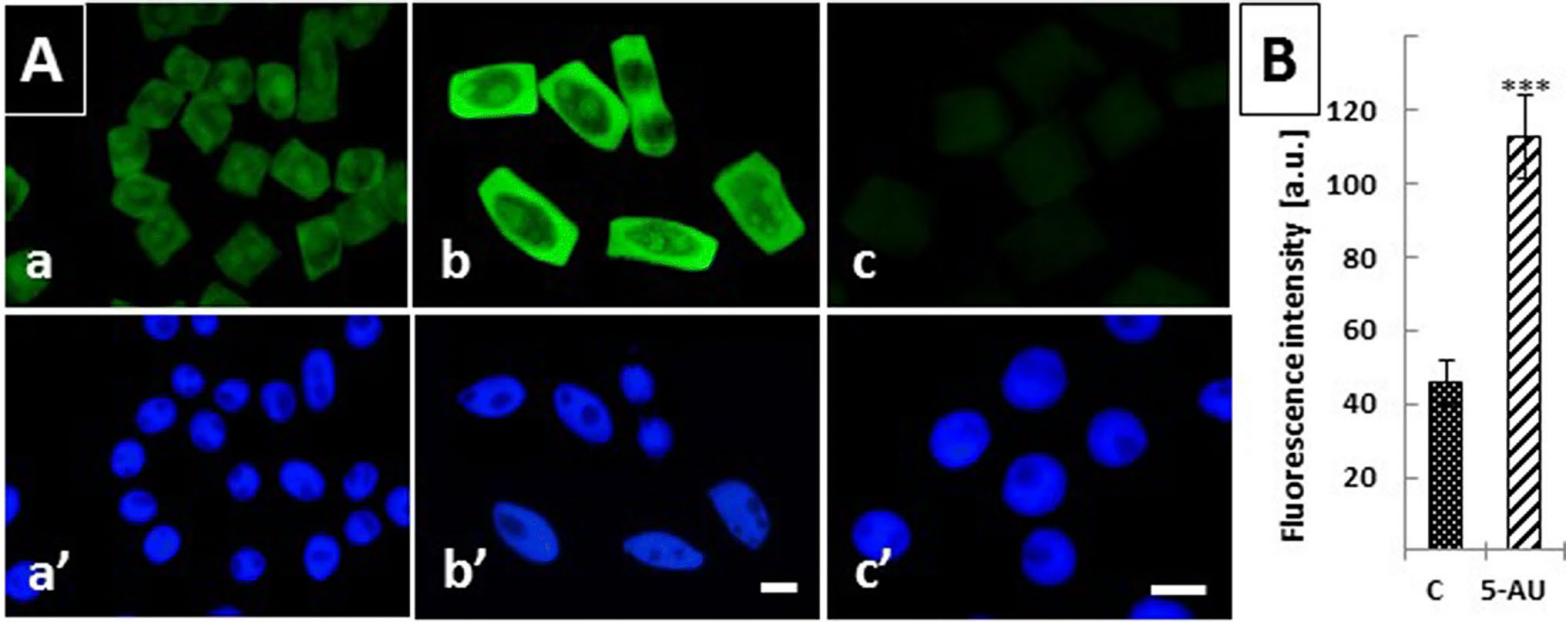
Nascent protein synthesis detected using Click-iT® HPG Alexa Fluor® 488 imaging in *A. cepa* RAM cells. (A) Immunofluorescence of proteins: control (a), 72 h treatment with 5-AU (b), negative control series performed using (50 µM) cycloheximide (c); corresponding images of the same cells counterstained with DAPI (a’–c’). Scale bars = 20 μm. (B) Microfluorimetric evaluation of protein synthesis levels (Fluorescence intensity [a.u.]) expressed in arbitrary units, calculated as mean pixel value (± SD) measured at the cytoplasm area. When compared with the control, the fluorescence intensity in 5-AU-treated is increased, with the statistical significance at *p* < 0.001

A similar reaction with L-homopropargylglycine (HPG), an amino acid analog of methionine, was used to determine an overall intensity of protein synthesis (Fig. 5A). The fluorescence measurements shown in Fig. 5B indicate that when compared with the control root cells (Fig. 5Aa, a), the translational activity of RAM cells significantly increases (by about 2.5 times) after 72 h treatment with 5-AU (Fig. 5Ab, b). No detectable HPG staining could be noticed in cells exposed to cycloheximide (a translation inhibitor in Eukaryotes), used as a specific negative control (Fig. 5Ac, c).

### 5-AU-induced H_2_O_2_ production, decrease in the reduced glutathione (GSH) level and induction DNA damage

**Fig. 6:**
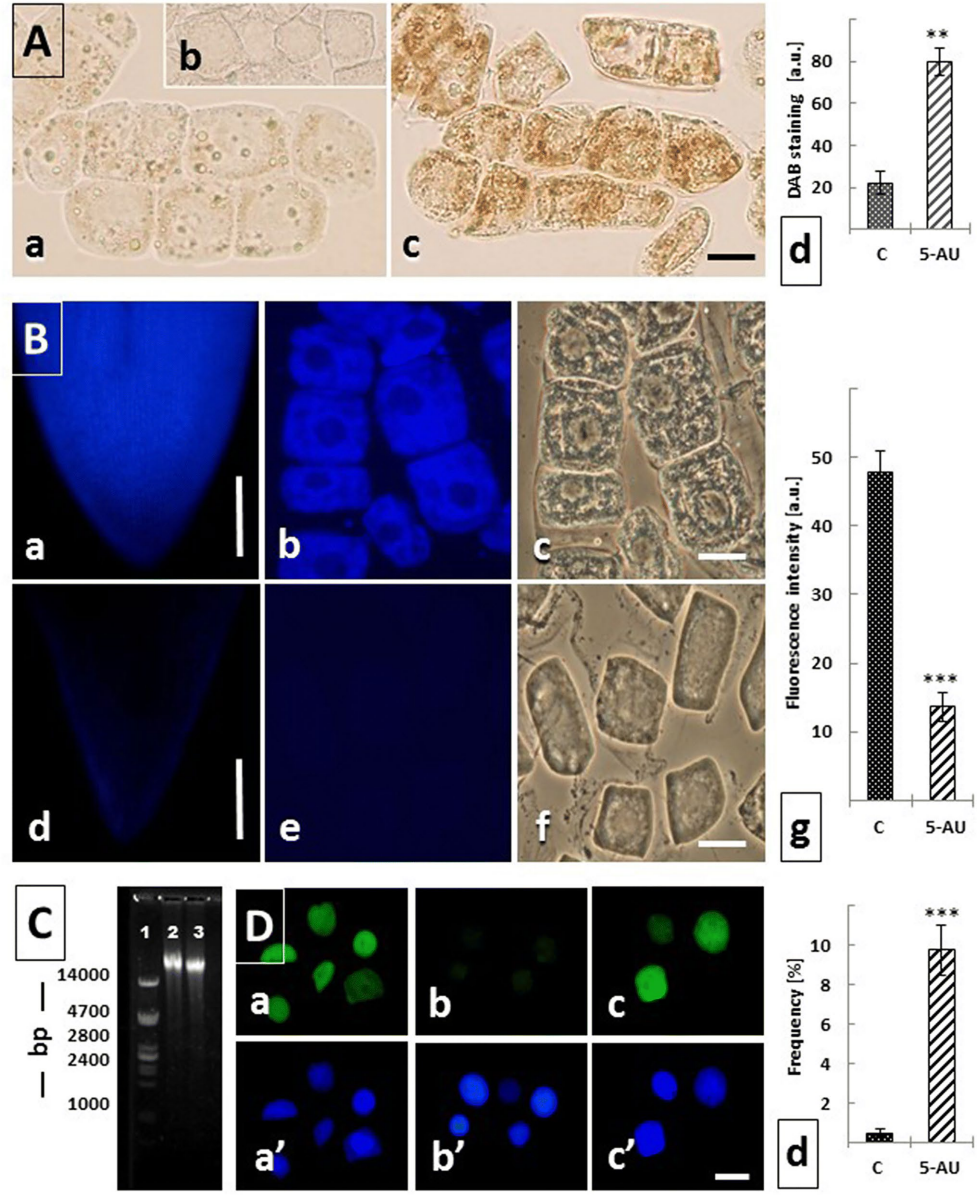
Generation of ROS, changes in GSH levels, DNA fragmentation, and induction of AL-PCD in *A. cepa* RAM cells following prolonged (72 h) treatment with 5-AU. (**A**) DAB-stained cells in the control root meristem (**a**), in root cells after 72 h treatment with the mixture containing 5-AU and 1 mM ascorbic acid (**b**), and in root cells incubated for 72 h with 5-AU (**c**). Scale bar = 20 μm. Cytometric evaluation of the DAB staining intensities in the control (**C**) and 5-AU-treated RAM cells ([a.u.]—arbitrary units); statistical significance at *p* < 0.001. (**B**) Detection of reduced glutathione (GSH) using ThiolTracker™ Violet fluorescence assay: longitudinal thick section of onion root meristems from the control (**a**–**c**) and 5-AU-treated seedlings (**d**–**f**); individual RAM cells at large magnification: control (**b**), 5-AU treatment (**e**), and the same cells viewed under phase contrast (**c**, **f**, respectively); scale bars = 500 μm for a and d, and 20 μm for (**b**, **c**, **e**, and **f**). Microfluorimetric evaluation ([a.u.—arbitrary units) of the mean total GSH fluorescence (± SD; g) in the control (**C**) and 5-AU-treated RAM cells, with the statistical significance at *p* < 0.001. **C** Agarose gel electrophoresis assay for DNA extracted and purified from onion root meristem cells: lane 1—mass marker, lane 2—control, and lane 3—5-AU treatment for 72 h. Note some shift of DNA towards lower buoyant density due to DNA fragmentation after incubations with 5-AU. **D** Detection of AL-PCD by TUNEL assay in DNase I-treated RAM cells (positive control; **a**), in the untreated control root cells (**b**), and in root meristem cells exposed to 72 h treatment with 5-AU (**c**); the same cells are visualized after DAPI staining (**a**–**c**), scale bar = 20 μm; mean frequency [%] of TUNEL-positive RAM cells in the control (**C**) and 5-AU-treated onion meristems; statistical significance at *p* < 0.001 (**d**)

Four types of analysis have been used to describe the damaging influence of prolonged (72 h) treatment with 5-AU on onion RAM cells (Fig. 6): (1) microcolorimetric evaluation of the hydrogen peroxide (H_2_O_2_) levels (Fig. 6A), (2) fluorometric estimation of the amount of reduced glutathione (GSH; Fig. 6B), (3) detection of AL-PCD by fluorescent visualization of cell nuclei containing free 3′-OH DNA strand breaks using TUNEL assay (Fig. 6C), and (4) DNA electrophoresis for the separation of different-sized DNA fragments (Fig. 6D).

Hydrogen peroxide (H_2_O_2_), one of the key reactive oxygen species (ROS), has a major first-line defense role under various biotic and abiotic stress conditions in plants. 3,3′-diaminobenzidine tetrachloride (DAB), which in the presence of peroxidase is converted into polymerized reddish-brown deposits (Thordal-Christensen et al. 1997), and was used to evaluate the H_2_O_2_ level in both control (Fig. 6Aa) and 5-AU-treated root meristem cells (Fig. 6Ac). The specificity of the reaction has been proved by incubating seedlings in 1 mM ascorbic acid (AA; H_2_O_2_ scavenger; (Fig. 6Ab). Microcolorimetric analysis revealed that after treatment with 5-AU, the mean intracellular level of H_2_O_2_ was significantly higher (about fourfold, at *p* < 0.002) than that observed in the control RAM cells (Fig. 6Ad).

The tripeptide thiol glutathione (γ-L-glutamyl-L-cysteinyl-glycine; GSH) is a powerful antioxidant and redox buffer involved in the detoxification of reactive oxygen species (ROS). The intracellular level of GSH was measured in the control (Fig. 6Ba–c) and 5-AU-treated root meristem cells (Fig. 6Bd–f) using glutathione detection reagent (ThiolTracker™ Violet), which reacts actively with reduced thiols groups in intact live cells. Although blue fluorescence staining appeared in both experimental variants, quantitative analysis clearly demonstrated that long-term exposure to low concentrations of 5-AU resulted in a statistically significant decrease (*p* < 0.001) in GSH level (Fig. 6g).

In order to evaluate the extent of DNA fragmentation, the agarose gel electrophoresis was used (Fig. 6C). When compared with the control (lane 2), a slightly increased mobility of relatively small DNA fragments extracted from 5-AU-treated RAM cells (lane 3) was observed.

The TUNEL assay (terminal deoxynucleotidyl transferase (TdT)-mediated dUTP nick-end labeling) combined with DAPI staining was used to detect cell nuclei containing free 3′-OH DNA strand breaks (Fig. 6D). Fluorescent-labeled nuclei were found in almost all cells treated with DNase I (positive control; Fig. 6Da, a). In contrast to untreated (control) onion RAMs, where only a small population of TUNEL-positive cells could be seen (about 0.5%; Fig. 6Db, b), after the 72-h treatment with 5-AU, the fluorescence signals were noted in about 10% of root meristem cells (Fig. 6Dc, c, Dd).

## Discussion

Chromatin organization and cell cycle progression are tightly interconnected, and a vast number of different conditions must be met at particular time intervals prior to and during mitosis in order to complete nuclear division. The wide-ranging changes of the nucleoplasm that occur throughout the cell cycle create two structural conformations, one adapted to transcribe and to faithfully replicate genomic sequences during interphase, and the other adjusted to distribute chromosomes into daughter cells at mitosis (Morales et al. 2001; Raynaud et al. 2014; Żabka et al. 2010, 2012). Both periods are commonly viewed as a continuous array of discrete stages, where each must be finished completely before the next one is allowed to start. To achieve this, the chronological sequence of metabolic events and successive step-to-step transitions is strictly monitored by signaling pathways known as the cell-cycle checkpoints (Hartwell and Weinert 1989).

Evidence from our previous studies (Żabka et al. 2010, 2012, 2013, 2015a, b) indicated that continuous treatment of onion RAMs with a low concentration of HU may disrupt crucial functions of the checkpoint response system, leading cells to abnormal mitotic progression manifested by premature chromosome condensation (PCC phenotype) and by axially elongated cells with half interphase/half mitotic structures (IM phenotype having both dispersed and condensed domains of chromatin). Our current work shows that prolonged incubations of 4-day-old primary roots of *A. cepa* with many chemical agents (including those commonly used to inhibit DNA replication) can induce interphase–mitotic organization of nuclear chromatin structures. Among all agents tested at nontoxic concentrations, 5-AU was found to be the most effective with regard to generating PCC and IM cells (with its effectiveness only slightly lower than that of HU).

There seems to be an evident discrepancy between the general phenotypic response of onion seedlings to 5-AU-induced stress conditions (applied at low concentration), manifested by the gradual reduction of root meristem size (observed also in all other experimental series), and the increased biochemical activities involved in DNA replication, transcription and translation. At present, it would be difficult to explain these conflicting results other than by assuming that—under the influence of harmful chemical substances or unfavorable environmental factors—certain plant-specific “problem-solving mechanisms” are organized and dynamically displayed to establish functionally effective and operationally efficient metabolic facilities, which help support the continued growth and differentiation of root cells and tissues.

In agreement with our earlier studies, both onion RAM cells with apparent symptoms of premature chromosome condensation (PCC-like cells), as well as those showing unusual biphasic IM nuclear structures should be considered as the ultimate consequence of three consecutive physiological events, taking place right from the start of the drug treatment: (1) an inhibitory stage induced directly by the DNA damage and the impaired DNA synthesis, (2) an adaptation stage comprising means to activate a complicated network of DNA damage response (DDR) mechanisms, and (3) a recovery stage, during which early symptoms diminish and some cells restore their lost functions despite the continued presence of the stress factor (Żabka et al. 2010, 2012, 2015a, b). As a consequence, the first observed effect of low-dose HU treatment in onion RAMs consisted in a decreased frequency of nuclear divisions, followed by a period of constant cell enlargement, which, in turn was succeeded by a renewed rise in mitotic activity, giving rise to various forms of mitosis, including PCC-like and IM cells. However, irrespective of this, what stands out as a common final outcome of all drug treatments, it seems like there is some kind of correspondence between the time-dependent effects observed in former experiments (confined to HU-generated DNA damage) and the succession of 5-AU-induced changes at the DNA replication level. Accordingly, our present results obtained using fluorescence EdU assays clearly indicate that, within the first 3 h of onion root treatment with 5-AU, the intensity of nuclear DNA labeling decreases (corresponding to the inhibitory stage), then, it increases continuously during the next several hours of incubation (adaptation stage), and afterwards, it attains the maximum values (2.6-fold higher than the minimum) at the 48–72 h time points (recovery stage). In fact, a similar timeline of changes, although not exactly matching, was obtained for EdU labeling indices, with their highest value recorded in *A. cepa* RAM cell populations as soon as after 12 h of treatment. The prolonged and partially overlapping periods of the enhanced intensity of EdU labeling and of the increased EdU labeling index suggest a large-scale reprogramming of DNA replication patterns, probably reflecting changes in origin concentration, in their efficiency, and in relative duration of DNA synthesis. Resolving these problems may come from a more precise characterization of the replicons using molecular combing of EdU-labeled DNA fibers, which seems feasible considering strong fluorescence signals obtained in our present simplified version of DNA extraction and spreading assay. Thus, it is probable that cell cycle checkpoints activation by low concentration of 5-AU causes additional firing of replication origins, modifications of DNA replication-associated proteins (De Piccoli et al. 2012), upregulation of dNTP pools (Jossen and Bermejo 2013), as well as relaxation of chromosome architecture to relieve topological stress (Bermejo et al. 2011).

It is well-known that collisions between RFs and transcription bubbles give rise to the formation of stable RNA:DNA hybrids (triple stranded structures termed R-loops), which slow-down or arrest DNA replication, induce premature termination of transcription, and generate DNA replication stress (Brambati et al. 2015; Gaillard et al. 2015; García-Muse and Aguilera 2016). Accordingly, spatial and temporal separation of replication and transcription can reduce the number of such conflicts, without preventing them completely, especially in large, transcriptionally active genes, characterized by an enhanced sensitivity to RS (Helmrich et al. 2011). Furthermore, a number of studies have shown that replication inhibitors can induce collisions between the transcription machinery and RFs, and affect the dynamics of transcription (Hamperl et al. 2017). It was hypothesized that the appearance of double-stranded breaks (DSBs) results in conflicts between replication and increased transcription at HU-induced genes (Hoffman et al. 2015). Hosseini et al. (2016) revealed that HU elicits a significant effect on the transcriptome of the murine embryos, where the expression of 1346 transcripts was significantly changed (at least 1.5-fold) in HU-treated embryos, when compared with the control group.

Although, as in the case of HU, the side effects of numerous forms of DNA damage reported in the present study result in a considerable decrease in RAM size, the response of root tip cells exposed to 5-AU is associated, paradoxically, with significantly increased activation of the transcriptional and translational expression levels. When considering the data from our experiments with EdU as a thymidine analog and the results obtained using 5-ethynyl uridine (5-EU) transcription assay, it is obvious that both DNA replication and transcription may perform their specific tasks efficiently and simultaneously using common DNA templates without mutual interference. Thus, irrespective of DNA mismatches formed by 5-AU located as a middle base of a triplex triad via Hoogsteen hydrogen bonds (Rana and Ganesh 2000; Shaker et al. 2016), the replication and transcription processes must have retained their ability to perform essential functions in a highly coordinated manner, preventing potential conflicts, which are the main intrinsic source of genome instability (Helmrich et al. 2011; García-Muse and Aguilera 2016). Correspondingly, it cannot be ruled out that the cellular response to 5-AU-induced RS includes rapid transcriptional activation of genes involved in DNA metabolism and in the mechanisms engaged in DNA damage repair systems.

Apart from the direct influence of 5-AU (and of other inhibitors of DNA synthesis) as the main source of DNA replication stress, there may be also an indirect influence, or multiplier effect, resulting from interactions between DNA and ROS, which are known to play a dual role of regulatory and toxic factors (Jajic et al. 2015; Mittler 2017). The oxidative stress results in a wide array of physiological and biochemical reactions, comprising disturbances in water balance, membrane injuries, reduced protein synthesis, changes in gene expression, alterations in the dynamics of metabolism and phytohormone levels (Gill and Tuteja 2010; Bita and Gerats 2013). If the state of stress lasts too long (or becomes intensified), it may lead to cellular disorders, tissue dysfunctions, and even to the death of the plant (Potters et al. 2009). Although the excess of free radicals, such as hydrogen peroxide (H_2_O_2_), hydroxyl radical (^·^OH), or superoxide anion (O_2_^·*−*^; Sharma et al. 2012; Demidchik 2015) can damage a variety of specific components within the genome, still little is known about the molecular mechanisms that protect DNA from oxidative stress. The attack of ROS on DNA results both in hydrogen abstraction from 2-deoxyribose (damaged sugar residues) and in addition of ^●^OH to the terminal carbon of the double bond (altered bases; Roldan-Arjona and Ariza 2009; Sharma et al. 2012). A link between inhibitors of replication and oxidative stress has been observed in several species, including HU-treated RAM cells in *Arabidopsis thaliana* (Yi et al. 2014) and endothelial cells with APH (Schilder et al. 2009).

The stress conditions in 5-AU-treated *A. cepa* RAM cells brought about a significant increase in the H_2_O_2_ concentration, which was evidenced using chromogenic DAB substrate. Similar effects, associated with the enhanced production of H_2_O_2_, have been observed in onion roots incubated with HU (Żabka et al. 2012), β-lapachone (Żabka et al. 2013) and sanguinarine (Żabka et al. 2017). Because H_2_O_2_ generates SSB, DSB (Żabka et al. 2013), and induce transcriptional activation of DNA stress genes, such as PARP2 and BRCA1 (Vanderauwera et al. 2011), it seems probable that aberrant chromatin morphology, formation of micronuclei, and induction of AL-PCD, are not only a consequence of a direct inhibitory influence of 5-AU, but also the effect of H_2_O_2_ (and other forms of ROS) generated as a secondary by-product induced by 5-AU. In consequence, the time-dependent activation of DNA damage response pathways and the ensuing resistance to stress conditions, accompanied by almost unchanged electrophoretic mobility of DNA, and a relatively small number of AL-PCD events may partially depend on a rapid increase in endogenous antioxidants levels. As evidenced, the stress–relief program of 5-AU-treated onion RAM cells is associated with a significant reduction in the intracellular level of the reduced glutathione (GSH), the dominant multifunctional thiol metabolite that plays a key role in plant growth and development and acts as the main ROS scavenger within plant cells during abiotic and biotic stress conditions (Zechmann 2014).

The data and observations reported in previous studies have shown that mild replication stress is correlated with: (1) an enhanced phosphorylation of H2AX histones (increase in number of γ-H2AX foci, which help recruit intra-S-phase checkpoint proteins and promote mechanisms of DNA repair), (2) changes in the progression of the S- and G2-phases, and (3) a gradual accumulation of cyclin B-like (CBL) proteins (Żabka et al. 2012). The asymmetrical accumulation of CBL proteins was implicated as a crucial factor responsible for the polarized activation of CDKs, and consequently, as a direct cause of an abnormal (IM) passage through the nuclear division. During this process, the onset of chromatin condensation is initiated in the vicinity of the pericentric regions, which was supported by the immunolocalization of phosphorylated H3 histones (H3S10Ph) at the most condensed segments of the emergent chromosomes (Żabka et al. 2010, 2012). Other prerequisites needed to facilitate the formation of IM cells were found to consist in: (1) a distinct separation of centromeric and telomeric domains of the interphase nucleus (known as Rabl configuration, which appears to be a consequence of the chromatin polarity acquired during mitotic division), (2) a parallel alignment of chromatin domains and the base → apex orientation of the RAM cell files, and (3) a specific association between the advanced mitotic (M)-poles of the IM cell nuclei and the polarized accumulation sites of PIN2 auxin efflux carriers and IAA (Żabka et al. 2015a). Our present experiments supplement the existing list of cellular conditions indispensable for the formation of both types of aberrant M-phase cells (having PCC-like or IM phenotypes) by including the enhanced transcription and translation levels, which allow for sustained nuclear functions and persistent cell growth. At the same time, they pose new problems for future research, concerning the mechanism by which stress conditions accelerate the dynamics of the DNA replication machinery.

## Abbreviations

5-EU: 5-Ethynyl uridine
AL-PCD: Apoptosis-like programmed cell death
APH: Aphidicolin
Ara-C: Cytosine arabinoside
DAB: 3,3-Diaminobenzidine tetrachloride
dNTPs: Deoxynucleotide triphosphates
DSBs: Double-stranded DNA breaks
EdU: 5-Ethynyl-2′-deoxyuridine
FLI: Fluorescent labeling intensities
FUdR: 5-Fluorodeoxyuridine
GSH: Reduced glutathione
H_2_O_2_: Hydrogen peroxide
HPG: L-homopropargylglycine
HU: Hydroxyurea
IM: Interphase–mitotic (cells, nuclei, or phenotype)
MTX: Methotrexate
PCC: Premature chromosome condensation
RAM: Root apical meristem
RFs: Replication forks
RNR: Ribonucleotide reductase
ROS: Reactive oxygen species
RS: Replication stress
SSBs: Single-stranded DNA breaks
